# Bringing NHS data analysis into the 21st century

**DOI:** 10.1177/0141076820930666

**Published:** 2020-07-16

**Authors:** Ben Goldacre, Martin Bardsley, Tim Benson, Kate Cheema, Roger Chinn, Ellen Coughlan, Sarah Dougan, Marc Farr, Loraine Hawkins, Adrian Jonas, Andy Kinnear, Morag Mcinnes, Mohammed Amin Mohammed, Caroline Morton, Rahul Pasumarthy, Neil Pettinger, Ben Rowland, Neil Sebire, Paul Stroner, Jeni Tennison, Samantha Warnakula, Oliver Watson, Emma Wright, Hamish Young, Jessica Morley

**Affiliations:** 1 DataLab, Nuffield Department Primary Care, Oxford OX2 6GG, UK; 2 Independent Consultant, previously 9058 The Health Foundation, London EC4Y 8AP, UK; 3 R-Outcomes Limited, Bristol BS49 4FS,UK; 4 5045 British Heart Foundation, London NW1 7AW, UK; 5 CCIO, 9762 Chelsea and Westminster Hospital NHS Foundation Trust, London SW10 9NH, UK; 6 Data Analytics Team, 9058 The Health Foundation, London EC4Y 8AP, UK; 7 London Borough of Islington Council, London N1 1XR, UK; 8 2241 East Kent Hospitals University NHS Foundation Trust, Kent and Canterbury Hospital, Canterbury CT1 3NG, UK; 9 Independent Consultant, previously NHS England, London SE1 6LH, UK; 10 National Institute for Health and Care Excellence, London SW1A 2BU, UK; 11 NHS South, Central and West Commissioning Support Unit, Oxford OX4 2LH, UK; 12 9664 Care Quality Commission, London SW1W 9SZ, UK; 13 The Strategy Unit – NHS Midlands and Lancashire Commissioning Support Unit, Kingston House, 438-450 High Street, West Bromwich B70 9LD, UK; 14 NHS NEL CSU, London EC2A 2DU, UK; 15 Kurtosis, Edinburgh EH6 6BZ, UK; 16 (Previously Avado Learning) Studio 3 Advisory, London SW16 1AJ, UK; 17 Great Ormond Street Hospital, London WC1N 3JH, UK; 18 Association of Professional Healthcare Analysts, Taunton TA3 5LY, UK; 19 Vice President and Chief Strategy Adviser, Open Data Institute, 65 Clifton Street, London EC2A 4JE, UK; 20 NHSX, London SE1 6LH, UK; 21 Bristol Health Partners, Bristol BS1 2NT, UK; 22 7047 Salford Royal NHS Foundation Trust, Salford M6 8HD, UK

Data have been widely hailed as ‘the raw material of the 21st century’^1^ and ‘better use of data’ is a central feature of the NHS Long Term Plan.^2^ Yet, data alone does not produce insights. To capitalise on opportunities to improve health and care, we need the data *and* outstanding data analysis. However, policymakers and academia have almost exclusively focused on pure academic research around the aetiology of disease; the field of practical coalface analytics has been largely neglected.

By practical coalface analytics we mean: variation in care analyses that identify opportunities to improve quality, safety and cost-effectiveness of care; modelling around waiting lists, or optimum locations for new services; evaluations of whether new interventions or reorganisations have achieved their clinical or logistic objectives; monitoring volume of activity and cost to ensure value from clinical contracts and more. These kinds of analyses are essential to ensuring data can be used to deliver improvements in patient care, earlier identification of problems and efficiency gains. They require similar skills, methods and tools to traditional epidemiology research. However, the practical analytics workforce is given little formal training and has been largely sidelined. Analyses are typically done behind closed doors, which blocks error-checking and reuse; clinicians and commissioners often lack the skills and support needed to ask good questions of data. Consequently, current use of data analysis to support decision making in the NHS is variable, and often poor.

To address these concerns we set out to: (i) identify the technical, cultural and regulatory barriers to the better use of analysis; (ii) identify potential solutions to these barriers; (iii) frame these barriers and solutions as action statements in a standard format (‘specific person/organisation s*hould do* this specific thing *so that* this specific outcome can be achieved.’); (iv) outline what successful change would look like in the format of ‘we'll know we’ve won *when*’ statements.

This paper reports the themes and solutions arising from our discussions. Specifically, we set out the need for: a 21st-century NHS analyst workforce supported by clear career trajectories and training opportunities; a culture of ‘build it once and share it to everyone’ built around modern, open analytic methods; capacity building for non-analyst staff to participate in conversations about data; and frameworks to ensure good value from externally commissioned analytics. We conclude by outlining the actions that NHS governing bodies can take to start making positive progress in these areas.

## A 21st-century NHS analyst workforce

The NHS currently has approximately 10,000 NHS data analysts.^2^ Most of these individuals are in very junior roles focused on data management which are advertised as ‘admin/clerical’ rather than ‘scientific/clinical’ and remunerated according to ‘Agenda for Change’ pay bands,^3^ which are constrained by requirements developed for nursing staff. Analysts are given little to no guidance on the skills that they need in order to progress, without strategic thought about providing inspiring leaders to look up to. This is compounded by the fact that, despite being highly technical and closely mirroring epidemiological research, operational research has evolved over time largely through informal sharing of methods among practitioners, rather than a formal literature or ‘commons of knowledge’. Combined, these issues pose a problem for career development and staff retention.

For the NHS, and its patients, to benefit from high-quality practical operational analytics, we need a 21st-century NHS analyst workforce, with a range of skills and skill levels, delivering innovative and efficient data analysis on questions relevant to clinicians, commissioners, patients and policymakers.^4^ To create this, NHS analysts need clear career trajectories and effective development and training opportunities.

### Clear career trajectories

To start, we need to recognise that if great analysts are to be retained in the NHS, they need to be inspired and to see opportunities for progression. Crucially, these opportunities should recognise and appropriately reward analyst's technical skills, which can have a high market value outside of medicine, whereas current NHS job descriptions require them to become generalist managers in order to rise in seniority. In our view, the NHS would do well to learn from other Government Analyst professions, for example: the Government Economic Service, Government Statistical Service, Government Operation Research Service and Government Social Research Service. These professions each have a head of profession, clear career paths and progression opportunities supported by genuine continuing professional development. They hold their staff to high standards by setting out clear best practice guidance, offering analysts accreditation and requiring analysts to adhere to a clear code of conduct.

To replicate this type of professional model, the NHS must start by formally reclassifying analyst roles under the NHS Agenda for Change categories as ‘scientific/clinical’, not ‘administrative/clerical’. This reclassification will require the creation of a new national competency framework and accompanying payscale that set out the job descriptions and skills required from junior analyst grades all the way up to a new head of profession role. This will make it clear to analysts what they need to do in order to progress, and help non-technical senior managers identify, appoint and train appropriately skilled data analysts. Looking further into the future, it is possible that a Royal College of Analysts (or equivalent) will be required. Such an institution can, if appropriate, look to develop methods of accreditation and licensing to re-assert professional identities and legitimise allocating resources for training. In the United States, for example, the American Medical Informatics Association has developed accreditation and certification standards for clinical informatics.^4^

### Training

Providing a clear progression pathway will only result in positive change if analysts are provided with the training they need to meet the skill requirements of higher grades. Training needs to be offered at different levels so that anyone who wishes to gain or improve their analytical skills can do so. For example: school leavers would benefit from the creation of an NHS Data Analyst and Data Scientist apprenticeship scheme; undergraduates and postgraduates would benefit from the creation of degrees in applied analytics for health and social care; and working professionals would benefit from accredited certificate programmes offering specialist skills training. Furthermore, those in the wider NHS workforce should be able to access training through massive open online courses in applied practical health analytics, so that all staff who wish to develop new and better analytic skills can do so. More data-literate managers will ask better questions of their analysts and be able to differentiate between good-quality and poor-quality work.

Clearly, the creation of these opportunities will not be ‘free’ either in terms of funding or staff time. For this reason, there will need to be a coordinated effort from national Arms-Length Bodies, local NHS organisations, national funders, the NHS Leadership Academy, Health Education England, academic organisations and others to provide leadership and funding. To justify this investment, those who benefit from NHS training schemes and from learning-on-the-job with NHS data must be expected to pay-it-forward and openly share their learnings. To facilitate this, those who are currently in senior management or analytical roles should be given time for capacity building; a platform to share from; and mentees.

## A culture of ‘build it once, share it to everyone’

Public trust in the use of NHS data relies heavily on transparency and accountability. Yet, it is currently difficult to hold people to account for poor quality analysis or for duplicative or wasteful analytic work. Complex analytic work in the NHS is commonly done in siloes, behind closed doors by national, local and regional NHS organisations, as well as private sector organisations. This means the results of the analyses are withheld from outsiders; critically, it also means that the methods used to process and analyse data are not shared. As a consequence of these closed working methods, people outside the direct analytic team are blocked from critically reviewing the methods to spot errors and fix them; nobody can learn from the work or replicate it; and nobody can reuse it on their local data. Furthermore the system is deprived of a commons of knowledge that would help train new and inspire staff, and provide the formal and informal structures needed to support collaborative improvement of analytic methods: it is notable that while most medical and paramedical specialties can fill several library shelves with textbooks, this is not the case for operational research in the NHS. In our view, adoption of modern, open analytic methods could rapidly build the collaborative culture that would support rapid innovation and capacity building.

### Use modern, open tools and approaches

As a starting point, the NHS needs to foster a culture that relies less on ‘manual labour in Excel’ and embraces the benefits of modern, open analytic methods such as re-usable scripts and open source tools including Python, R and Jupyter notebooks. This would benefit the existing analytic workforce and attract more highly trained data scientists, who are used to working this way, to work for the NHS. To begin, senior leaders should make it clear that these are acceptable methods for use within the NHS by: promoting, supporting and rewarding the use of open script-based tools; more actively supporting the use of platforms like Github and Stack Exchange; ensuring their staff have the time needed to share; providing best practice guidance on how to share appropriately; insisting that all shared analytical code is supported by ‘good enough’ documentation to enable reuse.

This will require a collective and modestly resourced effort to create a public library of tagged, edited and curated workbooks and ‘how-to guides’, with the patient data stripped out, that can be readily reused. Data controllers, regulators and policymakers can support this by making it mandatory (with exceptions) for NHS analysts to share code in this manner when the code has been developed with public resources. Furthermore, existing professional bodies, such as the Association of Professional Healthcare Analysts (AphA), should be supported to promote conversation and community around these shared resources by bringing the analyst community together twice a year for a conference (held on a weekday during work time and centrally funded) during which analysts can share insights, present work and build informal networks. Examples of great work showcased during these conferences should be written up in technical detail and added to the public library so that it can be celebrated and used to inspire others. Examples of where this is already happening are provided in Box 1. To minimise duplication, when analysts start a new project they should be expected to begin by identifying and evaluating existing solutions. To make shared scripts more widely reusable, central NHS organisations should aim to develop agreed standards for data schema wherever this is practical and desirable.

### Recognise the power of pooling technical skills and domain knowledge

Good data analysis is contextual. It is not just about knowing how to ask and answer a question using data, but knowing what the important questions to answer are and why, and how to interpret the answer in the context. Furthermore, what is best practice for data science in other fields might not be relevant or appropriate in the clinical domain depending on the specific features of medical data.^5^ Contextually specific analysis can only be delivered by teams that understand: where the data come from, its strengths and weaknesses; the right technical analytic approaches; how to communicate the outputs and how the outputs will be used to inform practical decisions in a system. As such, NHS analysts should not be isolated in analyst-only teams but embedded within mixed teams made up of analysts, clinicians, managers, researchers, software engineers and outstanding communicators. This will ensure user needs are better met and technical analysts are able to better understand the domain in which they are analysing data.

## Help staff become better customers and users of data

Managers and clinicians commonly feel out of their depth when commissioning or evaluating analytic insights provided to them. Conversely, analysts can feel frustrated by senior leaders asking unrealistic questions, or wanting to view numeric outputs as concrete ‘indicators’ rather than practical ‘measures’ to initiate a discussion. In addition, there is often no clear pathway or connection between data-driven research and real-world implementation. This leads to a disconnect between the user need and the analysis delivered. It is, however, possible to avoid this outcome.

As a starting point, NHS organisations should build an expectation that non-analyst staff will have sufficient data literacy to conduct informed conversations about data. To ensure this is not an unrealistic expectation, basic training in data analysis for clinicians and managers should be mandatory in training, and accessible (with adequate modest funds) later in career. National NHS bodies should hold local NHS bodies accountable for providing this training and enforcing staff attendance by commissioning a national, independently developed, ‘Analytical Capability Index’ to track whether an organisation has room to improve, and signal to leadership where gaps lie in their organisation, how they compare to peers, and who they can learn from.

### More thoughtful use of outsourcing

At present, analytic work is commonly outsourced, or commissioned from one NHS organisation by another (such as Commissioning Support Units). This may be driven by a lack of in-house capability for the reasons given above; it can also arise when NHS managers lack trust in their own analysts, or lack the technical capability to evaluate analysis conducted in-house. This skills shortfall at the commissioning level can result in weak product from the outsourced contractor, or a mismatch between aspiration and delivery. By giving managers the skills they need to better manage their own analysts, they will be capable of identifying when there is a genuine need to commission specialist input from health economists, epidemiologists or statisticians (for example), and when there is not. Furthermore, they will be better equipped to evaluate the outputs of commissioned analysis and to ensure that public money is well spent.

To further support this reduction in the reliance on more efficient and judicious use of specialist outsourced analysis, centralised support from a national advanced analytics advisory service should be provided. This support should include: standardised outsourcing contracts for analytics with clauses that all data and code are shared with the contracting NHS body; training and guidance on how to effectively commission – and then evaluate – external analytics; and appropriate procurement frameworks. Such a service could help govern the quality of outsourced analytics by requiring managers to get approval from it first before commissioning external analytics in the same way that the Cabinet Office governs government digital and technology spend.^6^ This can ensure that outsourced analytics adds value and that the process of outsourcing generates intelligence on specific skills gaps in the NHS. Lastly, local and national organisations should give careful consideration to the product expected from outsourced analytics: at present, a commissioning organisation will typically receive summary results without the full methodology used. This blocks critical evaluation and verification, but it also perpetuates the outdated closed approach critiqued above, at a time when we should be moving towards collaborative development of a commons of knowledge, as seen in all other areas of medicine and data science.

## Next steps

Delivering on the outlined suggested improvements will require collaboration across NHS organisations and the Government, as well as professional bodies, and detailed thought. However, we believe that the changes described are realistic and achievable, in part because many have previously been delivered in other sectors and countries. To enable delivery, we identified the following key domains where targeted action from specific NHS governing bodies could make a significant difference: promotion; training and professional development; knowledge sharing and skills exchange; community building; governance and standardisation; development of best practice. Collectively, for each of these domains, we developed targeted and practical ‘action statements’ in the format of: ‘this specific person/organisation *should do* this specific thing *so that* this specific outcome can be achieved’; the full list of ‘action statements’ informing the development of this paper is shared in the Supplementary Material. To maintain focus on tangible outcomes, we also broke down the overarching aim of ‘bringing NHS data analysis into the 21st century’ into tangible goals so that progress can be tracked. These are listed in Box 2 and take the format of ‘we'll know we’ve won *when* this specific outcome has been achieved’.

## Conclusion

There are huge opportunities for using data science to improve the quality, safety and efficiency of care. These opportunities are being needlessly neglected through a lack of clear career paths, and a historic failure to harness existing best practice into a commons of knowledge. But there is a vast skilled workforce that could, through use of open methods and structured support from the NHS, rapidly deliver an explosion in high-quality, verifiable, shared analytics. We hope this paper will stimulate further discussion between policymakers, analysts, the clinical workforce, data controllers and all members of the NHS and wider community, so that we can collaboratively achieve this goal.[Table table2-0141076820930666][Table table1-0141076820930666]

**Box 1. table1-0141076820930666:** Examples of good practice.

*NHS-R community* R is a powerful, free open source data science and statistics environment, used in industry, academia and major corporations (e.g. Microsoft, Google, Facebook) but its use in the NHS is almost non-existent. The NHS-R community, led by Professor Mohammed A Mohammed at the University of Bradford, aims to support the learning, application and exploitation of R in the NHS through workshops, video tutorials and providing a platform for discussion and sharing of developing best practice solutions to NHS problems. Find out more here: https://nhsrcommunity.com/ *Association of professional healthcare analysts (AphA)* AphA is a membership organisation which aims to raise the profile of healthcare analysts and provide a professional support network. It provides its members with a framework of professional standards that, along with standardised training and development opportunities, will ultimately lead to analysts becoming professionally registered. It also hosts an annual conference to foster community growth and knowledge sharing. Find out more here: https://www.aphanalysts.org/

**Box 2. table2-0141076820930666:** Short-, medium- and long-term goals written by workshop attendees in the format of ‘we'll know we've won when’ this specific outcome has been achieved.

*‘We'll know we've won when’*: • There are staff at board-level in managerial/strategic roles who once did an ‘inner join in Structured Query Language’, just as we have clinical leaders who once treated thousands of patients. • Problems are solved once by one Clinical Commissioning Group or Trust, who share their workbook to others. • Industry researchers and NHS analysts are able to learn from a rich competitive and collaborative ecosystem of analysts producing shared workbooks on a single library. • Github has more than 20 NHS analytics repositories with more than 100 contributors. • NHS analytic resources are deployed more on new interesting questions that can improve quality and safety of care, and less on financial monitoring. • Datasets and analytic approaches become more standardised because of collaborative, shared, transparent approaches. • Clinicians and managers know where to go to get answers, how to ask good questions and get good answers that meet their needs. • A manager who wants to learn how to be a better customer for data insights knows what to read and what course to go on. • An analyst who wants to help the NHS with their skills can find a course, and things to read, that help them develop their skills. • Your average sixth former will have heard of health analytics as a career option and where to learn more about it. • The NHS stops charging itself for exchanging raw data and only pays for value-added analytical activities. • There is a clear career pathway for analysts, including a specialist role that rewards and develops technical capability without recourse to becoming a manager. • Evidence-based decision making, backed by high-quality data analysis, is seen as business as usual across the NHS. • NHS analysts are seen as essential members of the workforce and are appropriately treated and rewarded. • There is a clear quality assurance process for data analysis conducted for the NHS by its own staff or by external consultants. • No claims such as ‘X Trust can save X millions by taking X action’ are made without the underlying methodology being made clear.

## Supplemental Material

sj-pdf-1-jrs-10.1177_0141076820930666 - Supplemental material for Bringing NHS data analysis into the 21st century Click here for additional data file. Supplemental material, sj-pdf-1-jrs-10.1177_0141076820930666 for Bringing NHS data analysis into the 21st century by Ben Goldacre, Martin Bardsley, Tim Benson, Kate Cheema, Roger Chinn, Ellen Coughlan, Sarah Dougan, Marc Farr, Loraine Hawkins, Adrian Jonas, Andy Kinnear, Morag Mcinnes, Mohammed Amin Mohammed, Caroline Morton, Rahul Pasumarthy, Neil Pettinger, Ben Rowland, Neil Sebire, Paul Stroner, Jeni Tennison, Samantha Warnakula, Oliver Watson, Emma Wright, Hamish Young and Jessica Morley in Journal of the Royal Society of Medicine

## Declarations

### Guarantor

BG

### Provenance

Not commissioned; externally peer-reviewed.

### ORCID iD

Jessica Morley https://orcid.org/0000-0001-5221-4770
