# Supplementary material for: Bringing NHS data analysis into the 21st century
Source: J R Soc Med. 2020 Jul 16;113(10):383–8. doi: 10.1177/0141076820930666 (PMC7575286; doi:10.1177/0141076820930666)
Supplement: sj-pdf-1-jrs-10.1177_0141076820930666 - Supplemental material for Bringing NHS data analysis into the 21st century [file sj-pdf-1-jrs-10.1177_0141076820930666.pdf]

Supplementary Material: Full list of action statements
Action statements written by workshop attendees in the format of *this specific person/organisation should do this specific thing so that this specific outcome can be achieved*

Promotion

- Ministers, shadow ministers and NHS spokespeople **should** reference NHS analysis and where it came from **so that** awareness of analytical work is raised among the public.

Training and Professional Development

- The NHS Staff Council **should** create a competency framework outlining what skills analysts need, and what their responsibilities will be, at each grade in Agenda for Change **so that** it is easier to recruit the appropriate staff and analysts have clear career progression opportunities
- The NHS Staff Council **should** re-classify analysts as scientific/clinical workforce, not admin/clerical in Agenda for Change **so that** more analysts are incentivised to join the NHS workforce and the accreditation and continuing professional development of analysts in the NHS is taken seriously
- NHS England **should** create heads of profession for senior analysts who have technical skills and mandate that these roles also exist at the local level **so that** high quality analysis is encouraged throughout the system and the NHS has to rely less on external consultants
- NHS managers **should** attend introductory courses in data analysis **so that** they can ask better questions of their analysts.
- NHS England **should** commission training on NHS data analysis from Health Education England **so that** we can develop the workforce.
- Health Education England **should** include NHS data analysis and flows as part of clinical training **so that** all clinicians can ask better questions and support analysts in meaningful interpretation of analyses.
- NHS organisations **should** provide protected work time and appropriate training budget to analysts for CPD, networking and shadowing opportunities **so that** analysts can build a professional portfolio and diverse contextual experience.
- Department of Health and Social Care **should** work with universities and fund the creation of new post-graduate certificates, diplomas, and degrees in applied practical analytics for health and social care **so that** the capacity of the NHS analyst workforce is increased
- Department of Health and Social Care **should** create an investment fund to support those who develop, deliver, share and curate analytics training **so that** NHS analysts have access to a rich range of continuing professional development opportunities
- NHS England **should** recruit volunteer senior analyst champions from across the system and give them resource for capacity building, a platform to share from and mentees **so that** junior analysts are encouraged to stay in the workforce
- The NHS Leadership Academy **should** create a graduate analyst scheme that parallels the NHS management graduate scheme **so that** junior analysts get exposed to different types of analysis needed in different parts of the NHS
- Health Education England **should** create an analyst pillar of its Technology Enhanced Learning programme that facilitates the creation of MOOCs (Massive

Open Online Courses) in applied practical analytics **so that** staff in all roles who wish to develop new and better analytic skills can do so easily
- Universities **should** invite local NHS analytical teams to careers events **so that** undergraduates are made more aware of career opportunities in health analytics.
- NHSX **should** produce an NHS analyst workforce strategy outlining how it will ensure analysts are given appropriate professional recognition, are remunerated appropriately and are encouraged to continue to develop **so that** analysts can make the best use of their skills and the NHS can recruit and retain excellent analytical staff.
- The GMC and Health Education England **should** ensure all undergraduate and postgraduate training for clinicians and managers includes knowledge of how data is captured and analysed to improve care **so that** the data literacy of the non-analyst workforce can be improved and the NHS is a better ‘customer’ of data analysis

Knowledge Sharing and Skills Exchange
- Academic researchers **should** consider closer partnerships with ‘coal face’ NHS analytical teams **so that** skills and knowledge exchange could be enhanced. This may include jointly funded University - NHS analytics posts.
- Government and central NHS analysts **should** spend time working in front line services **so that** they can better understand the issues and barriers in effective use of data throughout the service.
- NHS England **should** create guidance that makes it clear that modern open analytic methods such as re-usable scripts, open source tools such as Python, R, Juptyer, and open platforms like GitHub and Stack Exchange are not just permitted for NHS analysis use, but actively encouraged **so that** problems are fixed once for everyone, local reinvention of the wheel becomes unnecessary and reliance on manual labour in Excel is reduced
- NHS managers **should** ensure that their employees are given the time that they need to share their work **so that** duplication across the system can be reduced and efficiency improved
- Anyone commissioning or developing new analyses and tools **should** search for, and evaluate, existing solutions **so that** best practice can be highlighted and shared widely
- Funding bodies **should** (whenever possible) make sharing of any code developed with public resources as open source for re-use a contractual requirement for recipients of grants **so that** a commons of knowledge can be created
- A national **should** invest in creating an open library of “Great analytic work in the NHS” with technical documentation **so that** excellence is celebrated and the wider NHS analyst workforce can be informed
- NHSX **should** create an analysis assessment framework that, like the GDS (government digital services) service assessment framework emphasises the importance of multidisciplinary teams in creating outstanding services **so that** analysts are encouraged to work collaboratively with clinicians, researchers, software engineers and outstanding communicators with the aim of creating analysis that better meets users’ needs.

Community Building

- NHSX **should** pay for national conferences **so that** analysts can build a community and share insights.
- AphA **should** act as a lobbying and representative body for the analytical profession **so that** it has a voice at a national level.
- The Health Foundation **should** consider (in partnership with others) how to extend and deepen the analytical networks it has begun to support (for example using the model of the Q network and Q labs), **so that** the skills of the network can be leveraged for joint problem solving.
- NHSX **should** run national competitions for resources to solve problems around data extraction, transformation, analysis, interpretation and visualisation **so that** the development of an open, competitive, ecosystem of data analysis and community of NHS analysts can be fostered

Governance and Standardisation

- Royal Colleges, HQIP, NHS Digital and other relevant data controllers **should** make all data sharing agreements public and searchable **so that** others can benefit from example use cases and better understand how different data sources can be used.
- NHSX **should** include standards for data analysis in its Data Framework **so that** the quality of analysis is seen as, as important as the quality of underlying data.
- NHSX **should** create a cross-government health and care data analysis board **so that** other relevant and important sources of data (e.g. data re social determinants of health) can be identified and given due consideration.
- Department of Health and Social Care **should** found the creation of a 'Royal College' equivalent of NHS analysis **so that** standards of excellence can be maintained
- Health Education England **should** develop and promote an 'Analytical Capability Index' **so that** organisations that need to improve their analytical capabilities, can be identified and signposted to resources where they can learn from their peers
- NHS England **should** develop a 'code of conduct' for NHS data analysts **so that** analysts can be supported in the appropriate use, communication and interpretation of their outputs
- NHSX **should** create a national advanced analytics advisory service responsible for creating model contracts for NHS analytics consultancy and ensuring that all its own analytics is shared openly **so that** a more systematic approach to the procurement of external NHS analysis can be adopted and sharing practices are made more common and acceptable
- Department of Health **should** replicate the G-cloud model or 'approved suppliers' specifically for NHS data analysis **so that** public money spent on outsourced analysis is not wasted on poor quality outputs

Developing Best Practice

- NHS governing bodies **should** collaboratively agree model contracts for all out-sourced analytical work setting out the requirements for outputs, methodology, datasets and code, **so that** all analytical work meets set requirements of openness and re-usability.
- All analysts (internal and external) making claims such as "X Trust can save X Millions by taking X action" **should** publish their methodology and data **so that** the analysis can be reproduced and checked.

- Healthcare organisations such as NHS Trusts **should** support development of data and analytics infrastructure **so that** open, reusable tools and standards are in widespread use.
- CSUs and commercial vendors **should** only charge for analytic work that adds value to the data **so that** poor practices of ‘excel based manual labour’ that could be automated are discouraged.
